# Machine learning to identify heart failure with preserved ejection fraction in type 2 diabetes mellitus patients

**DOI:** 10.3389/fcvm.2026.1744588

**Published:** 2026-04-02

**Authors:** Bing-Yang Zhou, Cui-Ying Zhang, Ying-Yi Zhang, Ning Ren, Jie Geng

**Affiliations:** 1 Department of Cardiology, Chest Hospital, Tianjin University, Tianjin, China; 2 Department of Cardiology, Tianjin Chest Hospital, Tianjin, China; 3 Department of Cardiology, Tianjin Key Laboratory of Cardiovascular Emergency and Critical Care, Tianjin Municipal Science and Technology Bureau, Tianjin, China; 4 Clinical School of Thoracic, Tianjin Medical University, Tianjin, China

**Keywords:** diagnostic model, heart failure with preserved ejection fraction, machine learning, type 2 diabetes mellitus, XGBoost

## Abstract

**Aims:**

Type 2 diabetes mellitus (T2DM) is commonly observed in heart failure with preserved ejection fraction (HFpEF) patients. Despite its growing prevalence, HFpEF is frequently underdiagnosed. The aim of our study is to apply machine learning algorithms for identifying HFpEF in patients with T2DM.

**Methods:**

A total of 1,444 patients with T2DM who met the criteria were consecutively enrolled. Least absolute shrinkage and selection operator (LASSO) technique was applied for feature selection to identify key clinical variables. All patients were randomly divided into a training set and a test set at a ratio of 7:3. Extreme gradient boosting (XGBoost), random forest, K-nearest neighbors, support vector machine (SVM), light gradient boosting machine, decision tree and logistic regression were used to establish diagnostic models. The diagnostic performance of models was evaluated by the area under the receiver operating characteristic curve (AUC), precision, accuracy, F1 score, and Brier score. Calibration curve and decision curve analysis (DCA) were used to assess the accuracy and clinical validity of the model.

**Results:**

Patients were divided into HFpEF group and non-HFpEF group. XGBoost model (precision 0.812, accuracy 0.770, sensitivity 0.719, AUC 0.852, F1 score 0.741, Brier score 0.148) and SVM model (precision 0.784, accuracy 0.765, sensitivity 0.681, AUC 0.857, F1 score 0.745, Brier score 0.166) had the highest diagnostic performance. However, the calibration curve of the SVM model depart from the line of perfect calibration which confirmed poor calibration. Therefore, XGBoost was demonstrated to be best ML model in identifying HFpEF in patients with T2DM. Rankings of variable importance based on the Gain metric showed that B-type natriuretic peptide over 100 pg/mL had the highest importance score, followed by albumin, E/e′, age and high-sensitivity cardiac troponin T.

**Conclusions:**

This study found XGBoost to be the optimal machine learning algorithm in identifying HFpEF in T2DM patients. Additionally, the model demonstrated substantial clinical utility, as assessed by DCA.

## Background

Diabetes, a chronic metabolic disorder characterized by numerous comorbidities, is listed among the top eight leading causes of mortality and disability globally (1, 2). In 2021, there were 529 million people affected by diabetes worldwide, among which 95% were type 2 diabetes mellitus (T2DM) (3). By 2050, more than 1.31 billion people will be affected by diabetes. T2DM is commonly observed in heart failure with preserved ejection fraction (HFpEF) patients and may be an important factor in the pathogenesis of the disease (4).

According to the European Society of Cardiology (ESC) guidelines, HFpEF is a clearly defined clinical phenotype with the rising prevalence (5, 6). It was reported that HFpEF was present in nearly 70% of elderly patients over 65 years old with heart failure (HF) (7). A Chinese study analyzing data from 4,880 HF patients reported that 57% had HFpEF, with a five-year mortality rate of 13.4% (8). The Karolinska-Rennes (KaRen) study found that the 5-year mortality was 47% and 10-year mortality was high to 74% in HFpEF patients with acute HF (9). However, the diagnosis is difficult and challenged due to the heterogeneous characteristics and complex pathophysiology (10). HFpEF is always under diagnosed. A previous study investigating T2DM patients but without known HF reported that 22.9% were ultimately diagnosed with HFpEF (11). In addition, the prognosis of HFpEF is poor, with a 5-year survival rate of 35% reported in the literature (12). It has been demonstrated that diabetes was related to worse prognosis in HFpEF patients. In the TOPCAT trial, the phenogroup with obesity, diabetes and concentric left ventricular hypertrophy had higher risk of cardiovascular mortality, hospitalization for HF and cardiac arrest (13). The Dapagliflozin Evaluation to Improve the Lives of Patients with Preserved Ejection Fraction Heart Failure (DELIVER) trial enrolling HF patients with mildly reduced or preserved ejection fraction reported that the incidence of the primary outcome was the highest in the T2DM subgroup compared with the non-diabetes or prediabetes subgroups (14). Hence, it is imperative to identify individuals at high risk for HFpEF, enhance the early identification and improve the prognosis, particularly in patients with T2DM.

HFpEF can be diagnosed by invasive right heart catheterization, or estimated using several score systems including H2FPEF, HFA-PEFF, HFpEF-ABA. However, their practical application in clinical settings remains challenging. Machine learning (ML) is a rapidly growing field in cardiovascular medicine. Traditional regression methods have difficulty in capturing the complex data in large-scale datasets. However, ML is adept at harnessing large-scale datasets to model complex, non-linear relationships and higher-order interactions across multiple dimensions. Several studies have investigated the utility of ML in diagnosing cardiovascular diseases and predicting in-hospital and long-term mortality. Recently studies have used several ML algorithms to predict the severity of coronary artery disease and the risk of in-hospital HFpEF among patients with premature myocardial infarction (15, 16). In addition, ML was also applied to predict mortality in different HF populations (17–19).

Nevertheless, to date, the application of diverse ML algorithms for predicting HFpEF onset in patients with T2DM remains limited. Hence, the aim of our current study was to identify the risk of HFpEF in a T2DM population, utilizing multiple ML algorithms including extreme gradient boosting (XGBoost), random forest (RF), K-nearest neighbors (KNN), support vector machine (SVM), light gradient boosting machine (Light GBM), decision tree (DT), and blood biomarkers, echocardiographic data, demographic data. Our study further sought to compare the diagnostic performance of these ML models and traditional logistic regression to determine the most effective approach for HFpEF risk stratification in patients with T2DM.

## Study population

The current prospective study consecutively enrolled T2DM patients hospitalized because of chest tightness or shortness of breath between 1 March 2024 and 1 March 2025. Exclusion criteria were as follows: (1) LVEF <50%; (2) acute HF; (3) moderate and severe valve diseases; (4) congenital heart diseases, pericardial diseases; (5) severe renal dysfunction [estimated glomerular filtration rate (eGFR) below 15 mL/min/1.73 m^2^], severe hepatic impairment: Child-Pugh C; (6) advanced malignant neoplasm; (7) severe pulmonary diseases; (8) thyroid diseases. In addition, a total of 44 patients because of incomplete echocardiography data, 39 patients because of incomplete essential blood tests were not eligible in the initial screening of the study. The missing rate of each variable was less than 5%.

The diagnosis of HFpEF was established according to the 2021 ESC guidelines (5): (1) symptoms or signs of HF (e.g., dyspnea, lower extremity edema); (2) LVEF ≥50%, as assessed by experienced specialists in echocardiography using Simpson's method; (3) evidence of left ventricular diastolic dysfunction or elevated left ventricular filling pressure. Based on these criteria, patients were classified into two groups: the HFpEF group and the non-HFpEF group. Patients were diagnosed as HFpEF by their treating cardiologists according to guidelines mentioned above and subsequently reviewed by two senior heart failure specialists during patients hospitalized in our hospital.

A total of 1,444 patients with T2DM who met the criteria were included in the final analysis. The E/e′ ratio is defined as the early diastolic mitral inflow velocity (E) divided by the mean of the early diastolic tissue velocities (e′) measured at the lateral and septal mitral annulus.

This study adhered to the principles outlined in the 1975 Declaration of Helsinki. This study protocol was reviewed and approved by Tianjin Chest Hospital committee with the approval number (2024YS-004-01). All patients enrolled in the study provided written informed consent.

## Statistical analysis

Baseline characteristics were presented as mean ± standard deviation or median with interquartile range for continuous variables, and as frequencies with percentages for categorical variables. Differences between groups were assessed using the Student's t-test or Mann–Whitney U test for continuous variables, and the chi-square or Fisher's exact test for categorical variables. A two-sided *p*-value of <0.05 was considered statistically significant. The missing rate of each variable was less than 5%: hemoglobin data were missing in 4 patients, albumin data in 12 patients, and high-sensitivity cardiac troponin T (hs-cTnT) data in 5 patients. The median data was used to fill in the missing values for continuous variables. For categorical variables, multiple imputations were conducted. Importantly, the dataset with complete cases was established before splitting the data.

The feature selection process was informed by clinical experience and relevant diagnostic scores of HF. Least absolute shrinkage and selection operator (LASSO) technique was applied for feature selection to identify key clinical variables for the subsequent ML model and traditional logistic regression (LR) model. A total of 17 variables were included in our study. Multicollinearity among the independent variables was assessed by examining the tolerance and the variance inflation factor (VIF). VIF values greater than 5 were considered indicative of multicollinearity.

Six ML models and LR model were used to identify HFpEF in T2DM patients which included XGBoost, RF, KNN, SVM, Light GBM, DT. We used ten cross-validations to evaluate the performance of the ML models. The area under the receiver operating characteristic curve (AUC), precision, accuracy, F1 score, sensitivity/recall and Brier score were conducted to evaluate the performance of these models. F1 score combined precision and recall. Brier score, which measures the mean squared difference between the predicted probabilities and the actual outcomes, is used to assess the overall accuracy of the probability predictions. It ranged from 0 to 1. A higher F1 score, AUC, precision and accuracy while a lower Brier score indicated a better performance of the diagnosis model. Net reclassification improvement (NRI) and integrated discrimination improvement (IDI) were used to compare our diagnostic model and well-established scores. Differences in AUC between the models were assessed using the DeLong test.

To assess the accuracy and clinical validity of the model, calibration curve was used to present the agreement between model prediction and observed outcome. Decision curve analysis (DCA) was applied to quantify the net benefit for decision-making at different threshold probabilities. The net benefit integrates the benefits of true-positive classifications against the harms of false positives. The decision to initiate diagnostic examination or treatment for HFpEF depends on a probability threshold that balances the expected benefit of correctly identifying a case against the cost and risk of unnecessary procedures for false positives.

Statistical analyses were performed using SPSS software (version 27.0), R software (version 4.4.2) and JD_DCPM (V 6.11, Jingding Medical Technology Co., Ltd.). JD_DCPM software, developed using the R language, performs all statistical analyses through R code. It is designed as a code-free platform to offer a convenient statistical tool for medical researchers.

## Results

### Baseline characteristics

Baseline characteristics of the enrolled 1,444 patients were shown in Table 1. The average age of all eligible patients was 65.72 years. Patients in HFpEF group were statistically older than those in non-HFpEF group. HFpEF group had higher percentage of atrial fibrillation (AF), hypertension (HTN), but lower percentage of male. Left atrium (LA) was larger, and pulmonary artery pressure (PAP) and E/e′ were higher in HFpEF group. Patients in the HFpEF group were malnourished. They were characterized by significantly lower levels of hemoglobin (Hb) and albumin (Alb) compared with those in non-HFpEF group (both *p* < 0.001). No significant differences were observed between the training and test sets in terms of clinical characteristics, laboratory variables, echocardiogram, or electrocardiogram data. Moreover, the baseline characteristics between the training and test sets were considered well-balanced, as all standardized mean differences were below the threshold of 0.1 (Table 2).

**Table 1:** Baseline characteristics of patients in HFpEF group and non-HFpEF group.

Variables	Total (*n* = 1,444)	Non-HFpEF (*n* = 762)	HFpEF (*n* = 682)	*p* value	SMD
Clinical variables					
Age, years	65.72 ± 9.15	63.07 ± 9.05	68.69 ± 8.31	<0.001	0.646
Male, *n* (%)	863 (59.76)	477 (62.60)	386 (56.60)	0.020	0.123
AF, *n* (%)	84 (5.82)	3 (0.39)	81 (11.88)	<0.001	0.506
HTN, *n* (%)	1,004 (69.53)	497 (65.22)	507 (74.34)	<0.001	0.199
BMI, kg/m^2^	25.99 ± 3.47	26.21 ± 3.53	25.73 ± 3.39	0.009	0.138
Echocardiogram					
LVEF, %	60.39 ± 3.93	60.93 ± 3.74	59.79 ± 4.04	<0.001	0.294
LA, cm	0.38 ± 0.05	0.36 ± 0.04	0.39 ± 0.06	<0.001	0.610
PAP, mmHg	31.03 ± 4.72	30.05 ± 0.65	32.13 ± 6.66	<0.001	0.453
E/e′ ratio	9.61 (8.04, 11.84)	9.20 (7.73, 10.89)	10.20 (8.60, 12.66)	<0.001	0.435
Laboratory characteristics					
Hb, g/L	135.13 ± 17.93	138.07 ± 16.11	131.83 ± 19.25	<0.001	0.354
K, mmol/L	4.14 ± 0.37	4.14 ± 0.35	4.14 ± 0.39	0.932	0.001
Na, mmol/L	139.95 ± 2.25	139.94 ± 2.19	139.96 ± 2.31	0.877	0.008
Alb, g/L	41.80 (39.70, 44.00)	42.45 (40.50, 44.30)	41.10 (39.00, 43.50)	<0.001	0.424
eGFR, mL/min/1.73m^2^	89.74 (75.87, 97.40)	92.34 (80.75, 100.27)	86.56 (70.25, 93.70)	<0.001	0.468
Hs-cTnT, ng/mL	0.012 (0.008, 0.019)	0.010 (0.007, 0.015)	0.015 (0.010, 0.021)	<0.001	0.150
BNP, pg/mL	27.96 (10.00, 81.12)	10.78 (10.00, 20.90)	89.58 (46.96, 222.07)	<0.001	0.843
Electrocardiogram data					
P, ms	110.00 (102.00, 117.00)	111.00 (104.00, 117.00)	110.00 (100.00, 117.00)	0.010	0.248
PR, ms	169.00 (155.00, 186.00)	171.00 (158.00, 186.00)	167.00 (148.25, 185.75)	<0.001	0.371
QRS duration, ms	89.00 (83.00, 96.00)	88.00 (82.00, 96.00)	89.00 (83.00, 96.75)	0.088	0.131
QTc, ms	428.00 (412.00, 445.00)	424.00 (411.00, 439.00)	432.00 (415.00, 451.00)	<0.001	0.293

Data were expressed as mean ± SD, median with 25th and 75th percentile or *n* (%).

HFpEF, heart failure with preserved ejection fraction; SMD, standardized mean difference; AF, atrial fibrillation; HTN, hypertension; BMI, body mass index; LVEF, left ventricular ejection fraction; LA, left atrium, PAP, pulmonary artery pressure; Hb, hemoglobin; Alb, albumin; K, kalium; Na, natrium; Alb, albumin; eGFR, estimated glomerular filtration rate; hs-cTnT, high-sensitivity cardiac troponin T; BNP, B-type natriuretic peptide.

**Table 2:** Baseline characteristics of patients in training-set group and test-set group.

Variables	Total (*n* = 1,444)	Test-set (*n* = 434)	Training-set (*n* = 1,010)	*p* value	SMD
Clinical variables					
Age, years	65.72 ± 9.15	66.38 ± 9.03	65.44 ± 9.19	0.072	0.103
Male, *n* (%)	863 (59.76)	243 (55.99)	620 (61.39)	0.055	0.110
AF, *n* (%)	84 (5.82)	20 (4.61)	64 (6.34)	0.198	0.074
HTN, *n* (%)	1,004 (69.53)	310 (71.43)	694 (68.71)	0.304	0.059
BMI, kg/m^2^	25.99 ± 3.47	25.92 ± 3.53	26.01 ± 3.45	0.659	0.025
Echocardiogram					
LVEF, %	60.39 ± 3.93	60.49 ± 3.95	60.35 ± 3.92	0.542	0.035
LA, cm	0.376 ± 0.048	0.376 ± 0.047	0.376 ± 0.050	0.822	0.013
PAP, mmHg	31.03 ± 4.72	31.06 ± 3.72	31.02 ± 5.09	0.884	0.008
E/e′ ratio	9.61 (8.04, 11.84)	9.47 (7.98, 11.85)	9.66 (8.07, 11.84)	0.645	0.034
Laboratory characteristics					
Hb, g/L	135.13 ± 17.93	134.19 ± 17.86	135.53 ± 17.95	0.193	0.075
K, mmol/L	4.14 ± 0.37	4.13 ± 0.35	4.14 ± 0.38	0.860	0.015
Na, mmol/L	139.95 ± 2.25	140.05 ± 2.31	139.91 ± 2.22	0.272	0.064
Alb, g/L	41.80 (39.70, 44.00)	41.75 (39.60, 43.80)	41.90 (39.80, 44.10)	0.225	0.057
eGFR, mL/min/1.73 m^2^	89.74 (75.87, 97.40)	90.64 (78.01, 97.42)	89.53 (75.34, 97.39)	0.362	0.026
Hs-cTnT, ng/mL	0.012 (0.008, 0.019)	0.012 (0.008, 0.019)	0.012 (0.008, 0.019)	0.583	0.069
BNP, pg/mL	27.96 (10.00, 81.12)	28.16 (10.73, 79.05)	27.89 (10.00, 81.56)	0.602	0.089
Electrocardiogram data					
P, ms	110.00 (102.00, 117.00)	110.00 (102.00, 118.00)	110.00 (103.00, 117.00)	0.629	0.001
PR, ms	169.00 (155.00, 186.00)	170.00 (155.00, 189.00)	168.50 (154.00, 185.00)	0.150	0.144
QRS duration, ms	89.00 (83.00, 96.00)	89.00 (83.00, 96.75)	88.00 (83.00, 96.00)	0.195	0.090
QTc, ms	428.00 (412.00, 445.00)	429.00 (414.00, 445.00)	428.00 (411.00, 445.00)	0.406	0.043

Data were expressed as mean ± SD, median with 25th and 75th percentile or *n* (%). SMD, standardized mean difference; AF, atrial fibrillation; HTN, hypertension; BMI, body mass index; LVEF, left ventricular ejection fraction; LA, left atrium; PAP, pulmonary artery pressure; Hb, hemoglobin; Alb, albumin; K, kalium; Na, natrium; Alb, albumin; eGFR, estimated glomerular filtration rate; hs-cTnT, high-sensitivity cardiac troponin T; BNP, B-type natriuretic peptide.

### Models building and evaluation

**Figure 1:**
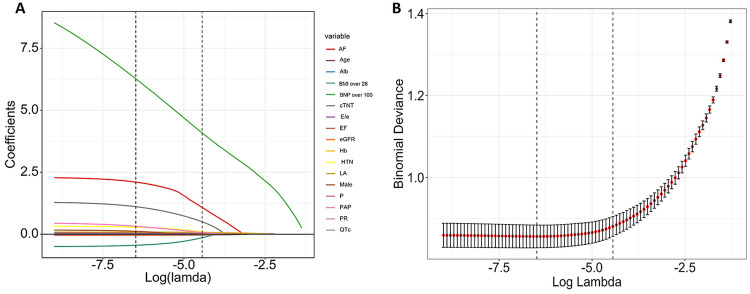
Least absolute shrinkage and selection operator (LASSO) binary logistic regression model for feature selection: coefficient paths **(A)**, cross-validation plot **(B)**.

All eligible patients were randomly divided into a training set (*n* = 1,010) and a test set (*n* = 434) at a ratio of 7:3. The training set was used to develop the ML models, whereas the test set was used to evaluate the predictive performances of the models. Predictor selection was performed using LASSO regression. Seventeen candidate variables were initially evaluated: AF, age, Alb, body mass index (BMI) over 28 kg/m^2^, brain natriuretic peptide (BNP) over 100 pg/mL, hs-cTnT, E/e′, left ventricular ejection fraction (LVEF), LA, PAP, eGFR, Hb, HTN, male, p wave in the electrocardiogram, PR interval, QTc interval (Figure 1A). A total of 13 variables were ultimately included in the final model (lambda.1se = 0.0118): age, AF, HTN, BMI over 28 kg/m^2^, LA, LVEF, PAP, E/e′, eGFR, Hb, Alb, BNP over 100 pg/mL, hs-cTnT (Figures 1A,B). All VIF values were below 5, indicating no significant multicollinearity among the 13 variables (Table 3).

**Table 3:** Multicollinearity among variables screened out by LASSO regression.

Variable	VIF
AF	1.346
E/e′ ratio	1.184
Age	1.637
Hb	1.373
eGFR	1.469
Alb	1.244
Hs-cTnT	1.026
BNP over 100 pg/mL	1.210
LA	1.414
LVEF	1.076
PAP	1.341
BMI over 28 kg/m^2^	1.135
HTN	1.066

Lasso, least absolute shrinkage and selection operator; AF, atrial fibrillation; Hb, hemoglobin; eGFR, estimated glomerular filtration rate; Alb, albumin; hs-cTnT, high-sensitivity cardiac troponin T; BNP, B-type natriuretic peptide; LA, left atrium; LVEF, left ventricular ejection fraction; PAP, pulmonary artery pressure; BMI, body mass index; HTN, hypertension.

**Figure 2:**
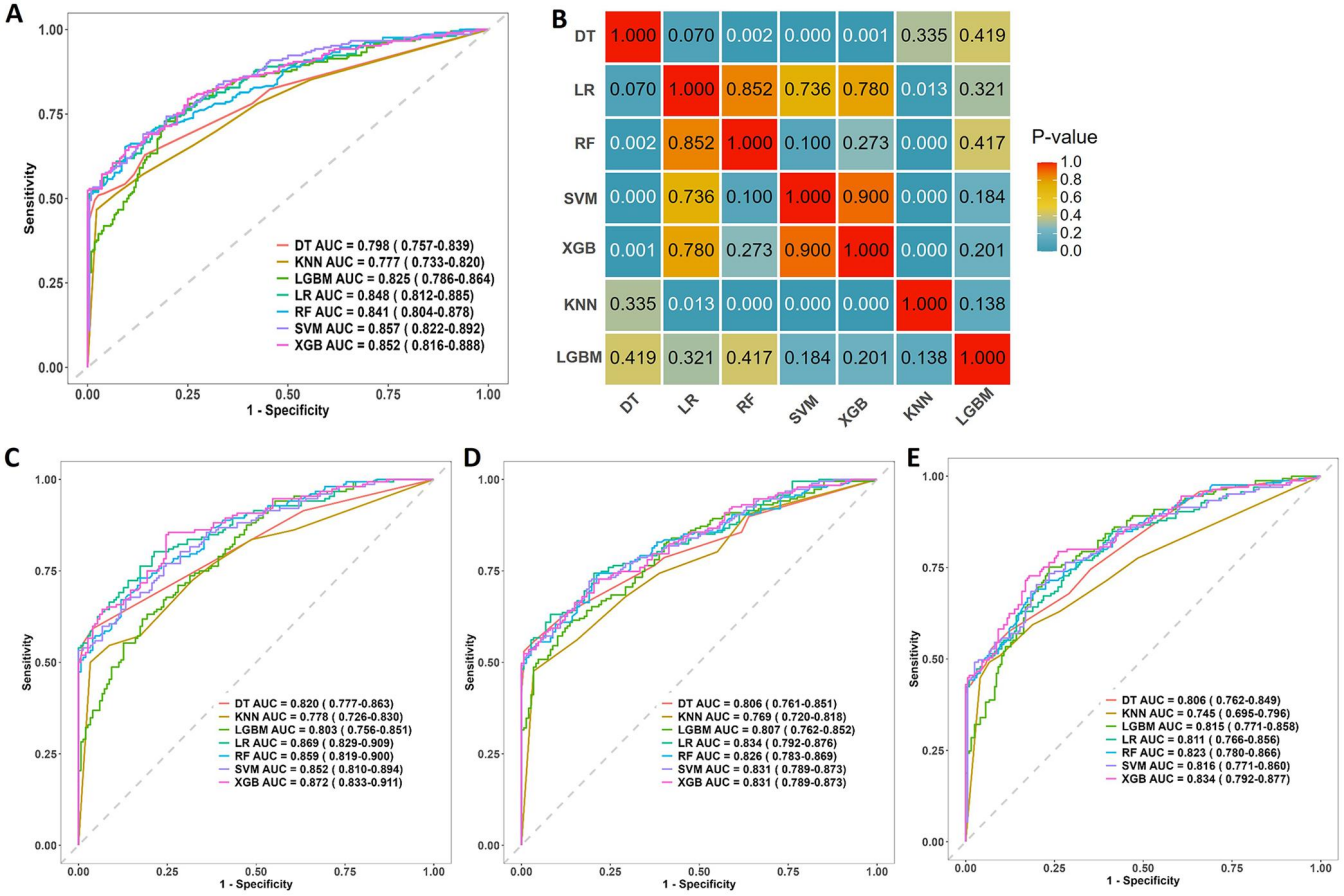
**(A)** the area under the receiver operating characteristic curve (AUC) of all models in the test set to predict the risk of heart failure with preserved ejection fraction (HFpEF) in patients with type 2 diabetes mellitus. **(B)** DeLong test among the AUC of the ML models. AUC of all models in subgroups. **(C)** hypertension subgroup; **(D)** subgroup of older population aged over 60 years; **(E)** coronary heart disease subgroup. DT, decision tree; KNN, K-nearest neighbors; LGBM, light gradient boosting machine; LR, logistic regression; RF, random forest; SVM, support vector machine; XGB, extreme gradient boosting.

**Figure 3:**
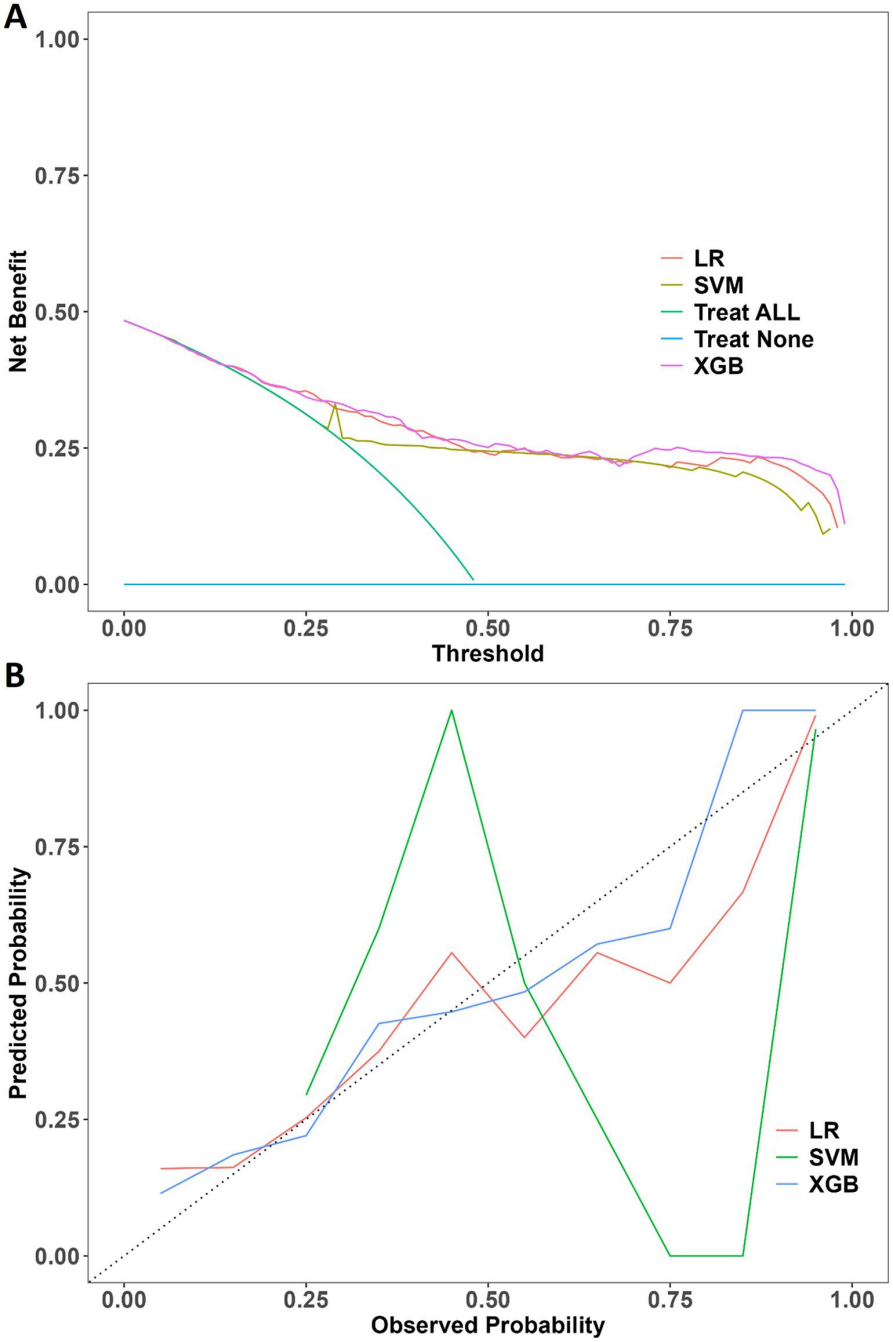
**(A)** Decision curve analysis (DCA) of the extreme gradient boosting (XGBoost), support vector machine (SVM) and logistic regression (LR) in the test set. **(B)** Calibration curve of the XGBoost, SVM and LR in the test set. LR, logistic regression; XGB, extreme gradient boosting; SVM, support vector machine.

**Figure 4:**
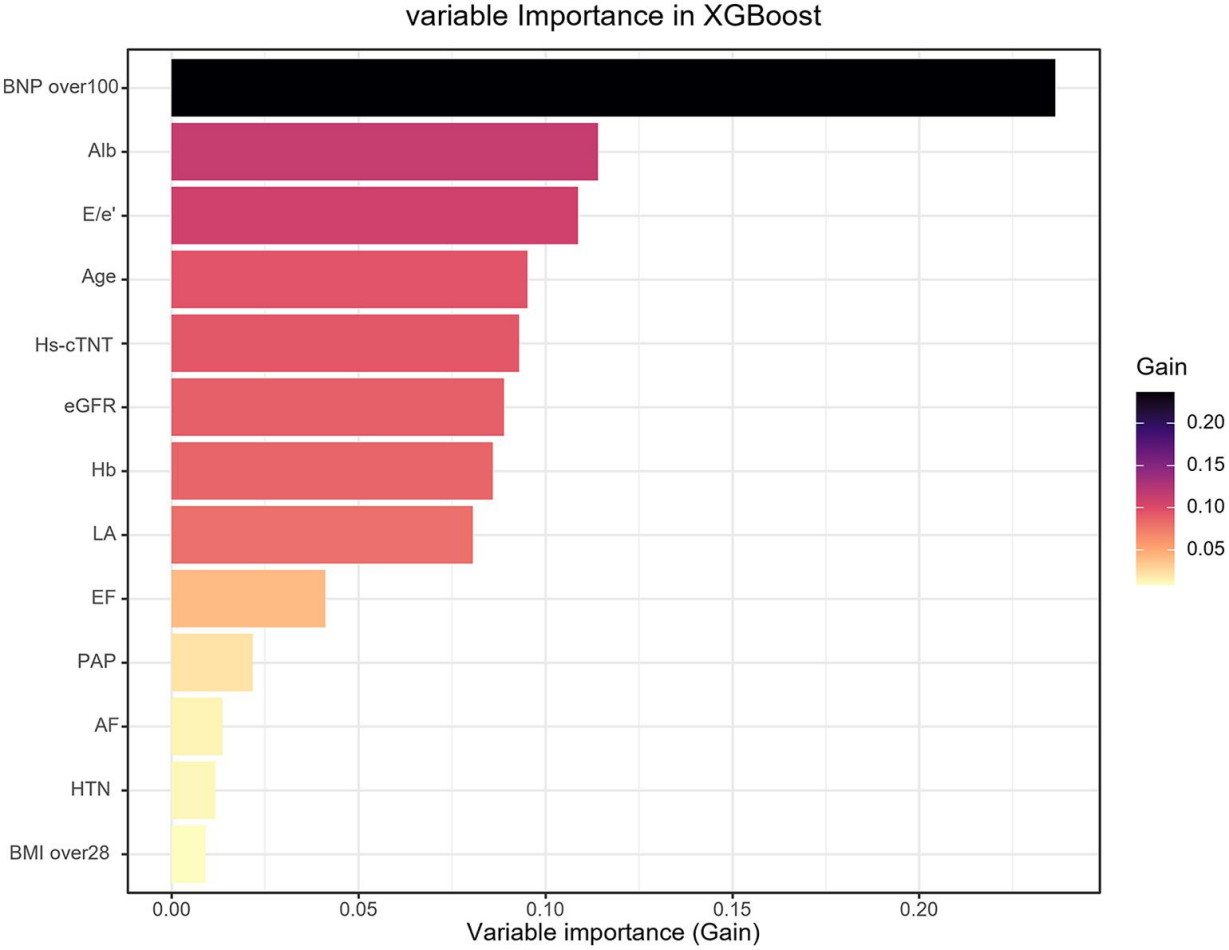
Rankings of variable importance based on the Gain metric in the extreme gradient boosting (XGBoost) model. BNP, brain natriuretic peptide; Alb, albumin; hs-cTNT, high-sensitivity cardiac troponin T; eGFR, estimated glomerular filtration rate; Hb, hemoglobin; LA, left atrium; EF, ejection fraction; PAP, pulmonary artery pressure; AF, atrial fibrillation; HTN, hypertension; BMI, body mass index.

The performances of all models were shown in Table 4. The receiver operating characteristic curves of all models were shown in Figure 2A. XGBoost model (precision 0.812, accuracy 0.770, sensitivity 0.719, AUC 0.852, F1 score 0.741, Brier score 0.148) and SVM model (precision 0.784, accuracy 0.765, sensitivity 0.681, AUC 0.857, F1 score 0.745, Brier score 0.166) had the highest predictive performance among all the models. Figure 2B showed the DeLong test among the AUC of the ML models. No statistical differences were found between the AUC of XGBoost and SVM. The DCA plot was employed to evaluate the clinical validity of XGBoost model and SVM model (Figure 3A). The net benefits of the test set for XGBoost model and SVM model were both significantly higher compared to the two extreme cases. The calibration curve of the XGBoost model aligned closely with the diagonal line of ideal prediction across all probability ranges (Figure 3B). In contrast, the calibration curve for the SVM model was notably unstable and departed from the line of perfect fit which confirmed poor calibration reliability (Figure 3B). Hence, XGBoost was demonstrated to be best ML model in predicting HFpEF in patients with T2DM. Figure 4 showed the rankings of variable importance based on the Gain metric which represents the average contribution of a feature to the model's predictive accuracy across all decision trees in the XGBoost model. Among these variables, BNP over 100 pg/mL had the highest importance score, followed by Alb, E/e′, age and hs-cTNT. XGBoost also demonstrated strong diagnostic performance in the subgroups of patients with hypertension, those aged over 60 years, and those with coronary heart disease (Figures 2C–E).

**Table 4:** Comparison of all models for predicting HFpEF in T2DM patients on the test set.

Model	Precision	Accuracy	Sensitivity/recall	AUC	F1 score	Brier score
XG Boost	0.812	0.770	0.719	0.852	0.741	0.148
SVM	0.784	0.765	0.681	0.857	0.745	0.166
DT	0.571	0.733	0.571	0.798	0.674	0.184
RF	0.807	0.772	0.688	0.841	0.747	0.154
KNN	0.701	0.698	0.657	0.777	0.678	0.227
Light GBM	0.780	0.744	0.671	0.825	0.713	0.170
LR	0.888	0.772	0.605	0.848	0.720	0.152

HFpEF, heart failure with preserved ejection fraction; T2DM, type 2 diabetes mellitus patients; ML, machine learning; HFpEF, heart failure with preserved ejection fraction; XG Boost, extreme gradient boosting; SVM, support vector machine; DT, decision tree; RF, random forest, KNN, K-nearest neighbors; Light GBM, light gradient boosting machine; LR, logistic regression.

We next compared our diagnostic model with H2FPEF score and ABA score (Table 5). The diagnostic model demonstrated a superior discriminative ability with a higher AUC when comparing to the two well-established scores (both DeLong test *p* < 0.01). Furthermore, it provided significant improvements in both risk reclassification and overall discrimination which were quantified by the NRI and IDI respectively.

**Table 5:** Comparison between the new diagnostic model with H2FPEF score and ABA score for predicting HFpEF in T2DM patients on the test set.

Model	NRI (95% CI)	*p* value	IDI (95% CI)	*p* value	AUC	DeLong test *p* value
H2FPEF score	–	Ref.	–	Ref.	0.780	–
Diagnostic model	0.294 (0.184–0.404)	<0.001	0.149 (0.100–0.203)	<0.001	0.848	0.001*
ABA score	–	Ref.	–	Ref.	0.754	–
Diagnostic model	0.233 (0.134–0.328)	<0.001	0.215 (0.161–0.269)	<0.001	0.848	<0.001**

HFpEF, heart failure with preserved ejection fraction; T2DM, type 2 diabetes mellitus patients; NRI, net reclassification improvement; IDI, integrated discrimination improvement; AUC, area under the receiver operating characteristic curve.

* Indicated DeLong test *p* value between the prediction model and H2FPEF score.

** Indicated DeLong test *p* value between the prediction model and ABA score.

## Discussion

Our study conducted six ML models and the traditional LR model to evaluate the best predictive model for HFpEF in T2DM patients. Several factors including precision, accuracy, AUC, F1 score and Brier score were used to choose the ML model with the highest predictive performance. XGBoost model presented good predictive performance and calibration curve. Additionally, the model demonstrated substantial clinical utility, as assessed by DCA. It was the first study to assess ML models for predicting HFpEF in T2DM patients.

ML enables systems to learn from autonomously from data without explicit programming. ML has been widely used in predicting the mortality in HF patients (20). A multicenter retrospective study enrolled 4,647 elderly patients with HTN and HF whose median age of 80 years and compared eight ML algorithms based on the AUC for in-hospital mortality prediction (18). The study reported that RF model had the highest performance, with the AUC of 0.850. They also used SHapley Additive exPlanations (SHAP) to analyze the impact of every variable on the final prediction result and found that urea, length of stay, neutrophils, Alb and high-density lipoprotein cholesterol were the most related variables for in-hospital mortality prediction. Another analysis from the Medical Information Mart for Intensive Care IV (MIMIC-IV) data demonstrated that the linear discriminant analysis (LDA) model presented the best performance to predict the all-cause mortality within 28 days with C-index of 0.813 in the test set (21). Moreover, the authors developed two dynamical online calculators based on the LDA model and the nomogram model. Another study applying five ML approaches reported that XGBoost model provided the highest discrimination and the lowest Brier score in predicting three-year mortality (22). In a study of patients with acute myocardial infarction (AMI) comparing seven ML approaches for predicting HF, XGBoost was identified as the optimal model with an AUC of 0.966 (23). This model maintained an AUC of 0.94 in external validation.

Consistent with the previous studies, our study also found that XGBoost model had the highest diagnosis performance. In addition, we also found that the calibration curve of this ML model aligned closely with the line of perfect calibration, indicating a strong agreement between the predicted probabilities and the observed risks. ML algorithm can integrate various variables such as demographics, laboratory results, imaging and then build models for clinical diagnosis or prognosis (24). XGBoost has advantages in clinical prediction which can be partly attributed to its technique characteristics (25–27). It is known that there exhibit non-linearity and complex interactions among clinical risk factors. XGBoost algorithm has the characteristics of identifying variables interactions and regularization technique which can significantly prevent overfitting and ensure the generalization ability on the test set. In addition, the ranking of variables importance analysis of the model may remind clinical scholars the potential unknown predictors. As mentioned in the result, the calibration curve of SVM showed some deviation from the ideal line. Firstly, the cause is that SVM algorithm is intrinsically designed for optimal class separation rather than probabilistic prediction. The *post-hoc* process to probabilities may increase the instability of the model. Secondly, mixed variable types including categorical and continuous variables in our dataset also posed challenges for the performance of SVM algorithm in handling probability-relevant interactions.

Studies in predicting the risk of HFpEF were limited. Recently, a study conducted five ML models in AMI patients and explored the performances of these models in in-hospital HFpEF risk prediction (16). The study identified XGBoost as the best ML model with an AUC of 0.854 and Brier score of 0.143. Age, BNP, HTN and BMI were finally included in those ML models and a visual prediction system. Many risk factors such as age, female, obesity, AF and metabolic syndrome contributed to the occurrence of HFpEF (28). H2FPEF score, a simple and clinically convenient method to diagnose HFpEF, includes variables of HTN, AF, obesity, age and echocardiographic elements (29). HFA-PEFF diagnostic algorithm includes BNP and several echocardiographic indicators such as E/e′ and PAP (30). HFpEF-ABA score includes age, BMI and AF. These tools integrate echocardiographic and clinical markers to estimate the likelihood of HFpEF. The H2FPEF score focuses more on disorders of cardiovascular metabolic diseases with less echocardiographic data. The HFA-PEFF score relies more on complex numerical values from echocardiography, while the data is challenging to obtain in some hospitals. HFpEF-ABA is easy to conduct clinically, while ignores some objective examination indicators. Moreover, these scores depend on traditional algorithm and more cardiac-specific data. Nutritional status is an important factor that cannot be ignored in patients with HF. Our model captured several factors included in the diagnostic scores mentioned above as well as nutritional factors such as Hb and Alb. Additionally, our study leveraged ML to develop a predictive model specially in the high-risk population of patients with established T2DM.

Another biomarker hs-cTnT was also demonstrated to be significantly associated with adverse events in patients with HFpEF. Patients with higher hs-cTnT levels had lower event-free survival rates (31). Aging has become a serious problem worldwide. Cellular aging promotes oxidative stress, myocardial remodeling and microvascular endothelial dysfunction, resulting in left ventricular (LV) impairment (32, 33). The incidence of HF increases with age. Epidemiological study in China estimated that the prevalence of HF among people aged 65–79 was 3.86%, and among those aged 80 and older, it was 7.55% (34). Similarly, the costs of the elderly patients were higher. It was reported that patients with HF over 65 years old showed 1.6-time higher costs than the young in South Korea (35). The prevalence of AF in HFpEF was reported as 40%–60% (36). AF increased biatrial volumes, while decreased LA compliance, LA reservoir strain and global longitudinal strain. Therefore, the coexistence of AF and HFpEF is associated with a worse prognosis. Malnutrition might be related to HFpEF. A study from Japanese HFpEF patients found that anemia predicted adverse events (37). An independent association was observed between Hb level and all-cause mortality among HFpEF patients.

The current study had several limitations. Firstly, it was a single-center study and the results were not validated in external data. Secondly, though we conducted stratified 10-fold cross-validation for hyperparameter optimization and bootstrapping on the test data to prevent overfitting, the sample size of our data was relatively small when comparing with other studies. External validation in a larger and multi-center population would enhance the credibility of the model. Thirdly, the biomarkers evaluated in this study were limited to demographic, echocardiographic, and laboratory data. The predictive performance in clinical practice could be further improved by adding electrocardiographic and echocardiographic imaging through ML or deep learning approaches. In addition, a hybrid approach combining clinical knowledge with data-driven feature selection was also needed for screening out novel, non-traditional indicators in subsequent study.

## Conclusions

In this study, we compared the performance of seven models in identifying HFpEF in T2DM patients using Chinese cross-sectional data. XGBoost was demonstrated to be the superior model with high predictive efficacy and clinical benefit. This study may provide a useful method for the identification and early management of HFpEF in patients with T2DM.

## Data Availability

The original contributions presented in the study are included in the article/Supplementary Material, further inquiries can be directed to the corresponding author/s.
